# Optimization of Ultrasound-Assisted Extraction of Anthocyanins from Torch Ginger

**DOI:** 10.3390/foods15030450

**Published:** 2026-01-27

**Authors:** Menuk Rizka Alauddina, Viki Oktavirina, Widiastuti Setyaningsih, Mercedes Vázquez-Espinosa, Miguel Palma

**Affiliations:** 1 Department of Analytical Chemistry, Faculty of Sciences, Institute for Viticulture and Agrifood Research (IVAGRO), University of Cadiz, Agrifood Campus of International Excellence (ceiA3), 11510 Puerto Real, Cadiz, Spain; menukrizkaalauddina@gmail.com (M.R.A.); miguel.palma@uca.es (M.P.); 2 Department of Food and Agricultural Product Technology, Faculty of Agricultural Technology, Gadjah Mada University, Jolan Flora No. 1, Bulaksumur, Sleman, Yogyakarta 53281, Indonesia; viki.o@mail.ugm.ac.id (V.O.); widiastuti.setyaningsih@ugm.ac.id (W.S.)

**Keywords:** edible flowers, torch ginger, ultrasound-assisted extraction, anthocyanins

## Abstract

The growing interest in using edible flowers as functional ingredients has increased the demand for reliable and sustainable strategies to recover and characterize their bioactive compounds. Torch ginger is a tropical species rich in anthocyanins. In this study, an ultrasound-assisted extraction (UAE) method was developed, optimized, and validated for the efficient recovery of anthocyanins from torch ginger flowers, with a clear focus on food-related applications. A Box–Behnken experimental design was applied to evaluate the influence of solvent composition, temperature, solvent-to-sample ratio, and pH on anthocyanin yield, using chromatographic responses. Solvent composition and solvent-to-sample ratio were identified as the most influential parameters, and effective extraction was achieved under mild temperature and pH conditions. The optimized conditions consisted of 84% methanol in water as the extraction solvent, a temperature of 30 °C, a solvent-to-sample ratio of 20:1 (mL g^−1^), and a pH of 5.6. Kinetic studies revealed that a 5 min extraction time maximized recovery while preventing compound degradation. The method was successfully applied to different torch ginger varieties, revealing a strong correlation between flower color and anthocyanin concentration. This research provides a fast, reliable, and environmentally friendly approach for assessing anthocyanin content in torch ginger flowers. The results support the valorization of this edible flower as a potential source of natural colorants and bioactive ingredients, contributing to ingredient selection, quality control, and the future development of functional foods and clean-label products.

## 1. Introduction

Edible flowers have been consumed for thousands of years in many European and Asian countries [1], where they have been used as flavor enhancers in many dishes and infusions, and as aesthetic elements, influencing the taste and appearance of dishes [2,3]. Nowadays, their culinary use has expanded due to their ability to add aroma, color, and texture to foods, with the torch ginger flower being particularly noteworthy, as it is traditionally consumed as a vegetable. This flower is an herbaceous plant belonging to the *Zingiberaceae* family, and is native to Sumatra (Indonesia) [4]. Its inflorescence shows eye-catching and attractive colors that vary from red and pink to white, and the flower is an essential part of various traditional dishes consumed in Malaysia as a spice and condiment, either raw or cooked [5], in Thailand as a vegetable and salad [6], and in Indonesia as a complement to typical dishes [7].

Besides culinary purposes, torch ginger has attracted growing scientific interest due to its nutraceutical properties, potentially being usable as a source of medicine due to its availability and its ability to be developed into a plant-derived drug [8]. Secondary metabolites from this plant, including phenolic acids, flavonoids, glycosides, saponins, tannins, steroids, terpenoids, and anthocyanins, have revealed their pharmacological effects, including antihyperglycemic, anti-inflammatory, antimicrobial, antioxidant, and antitumor activity [9,10].

It is particularly notable for its anthocyanin content. Anthocyanins are a class of polyphenols belonging to the flavonoid family, and are responsible for the wide range of colors found in flowers, fruits, vegetables, and other plants [11]. Their structural diversity, determined by the number of hydroxyl groups they contain, the position and number of sugars attached to the molecule, the number of aromatic acids attached to the sugars, and their pH-dependent behavior, influences their stability, functionality, and coloration, increasing their relevance in both nutrition and food technology [12,13]. Furthermore, these compounds play an essential role in human health. Their dietary intake has been linked to a reduced risk of chronic degenerative and cardiovascular diseases due to their ability to reduce oxidative stress in the body. Numerous studies have shown that anthocyanins display a range of biological activities, including antioxidant [14], anti-cancer [15], anti-diabetic [16], and antibacterial properties [17], which, together with the growing demand for natural colorants and safer alternatives to synthetic compounds, are driving their study and valorisation.

Edible flowers have the potential to become a part of the functional food industry to help in the stabilization of foods and lengthening their shelf lives, which makes the analysis of their anthocyanin content very interesting [18]. In this context, the characterization of anthocyanin content in edible flowers such as torch ginger is not merely an analytical challenge, but a key step toward their valorization within the agri-food chain. Knowledge of anthocyanin levels and profiles can guide the selection of suitable flower varieties, inform processing strategies, and support the development of novel food products enriched with natural pigments and bioactive compounds. Moreover, understanding how extraction conditions influence anthocyanin recovery is crucial for designing scalable, sustainable, and cost-effective processes compatible with food industry requirements. Additionally, edible flowers are well-accepted by consumers worldwide due to globalization and health awareness, which contribute to the improvement and resurgence in healthier lifestyles [1,10].

Extraction is an essential step in recovering, identifying, and quantifying anthocyanins from plant matrices, the study of which requires rapid, reliable, efficient, and environmentally friendly methods [19]. Since anthocyanins are soluble in polar solvents, they can be extracted from plant materials using methanol that is slightly acidified with either formic or hydrochloric acid. The acid reduces the pH of the solution, preventing the non-acylated anthocyanin pigments from degrading [20]. In addition, solid samples must be ground and homogenized beforehand, making it easier to take a representative sample for analytical-scale extraction [21]. Conventional extraction methods, such as maceration and the Soxhlet method, have some limitations regarding their high solvent consumption, which contributes to increased costs and creates additional environmental concerns, as well as the prolonged extraction times required for extraction, which can lead to the thermal degradation of the compounds [22]. These disadvantages have driven the development of advanced extraction techniques. In the case of anthocyanins, their polar nature requires the use of acidified solvents and procedures that minimize their degradation, making the choice of extraction method a critical factor in obtaining representative and reproducible results [23].

Among emerging technologies, ultrasound-assisted extraction (UAE) has been reported as a “clean” extraction method in terms of low solvent and energy consumption, short extraction time, high efficiency and yield, and low cost, and it has been widely used to extract bioactive compounds from natural plant resources [24,25]. Vinatoru highlights that this sonication method increased mass transfer intensification and improved solvent penetration into plant tissue, favoring the extraction of targeted compounds [26]. The UAE method has been reported as a better method for thermolabile components, such as anthocyanins, as it can operate at moderate temperatures, reducing the oxidation and hydrolysis effects observed in traditional methods [27]. Consequently, UAE is presented as a particularly promising technique for the rapid and effective extraction of anthocyanins from flowers, strengthening its potential use in food and functional applications. Variables such as sonication power, frequency, extraction time, solvent-to-sample ratio, pH, solvent composition, cycle, and extraction temperature directly influence the extraction yield, so proper optimization is key to obtaining high-quality extracts [28].

The determination and quantification of extracted anthocyanins can be achieved using both spectrophotometric and chromatographic techniques, each with specific advantages depending on the level of detail required. UV–Vis spectrophotometric methods, based on Lambert Beer’s law, allow for a quick, easy-to-use, and low-cost estimation of the total anthocyanins present in the sample thanks to their high absorption at a maximum wavelength between 510 and 550 nm, which is outside the range of other phenolic compounds [21,29]. However, when it is necessary to identify and quantify each anthocyanin individually, liquid chromatography is preferred due to its high selectivity, sensitivity, resolution, and sample behavior [30,31]. According to the literature, several anthocyanins have been identified in torch ginger flowers, with cyanidin-3-glucoside being the predominant compound [32]; therefore, the use of a chromatographic method that allows the individual determination of this anthocyanin is recommended.

The present study aims to develop and validate an ultrasound-assisted extraction method for the efficient recovery of anthocyanins from torch ginger flowers, with a clear focus on applications in food science and technology. By optimizing key extraction parameters using a response surface methodology and chromatographic evaluation, this work seeks to provide a rapid, reliable, and environmentally friendly tool for assessing anthocyanin content. Ultimately, the results contribute to the characterization and valorization of torch ginger flowers as promising natural sources of colorants and functional ingredients, supporting their future incorporation into food products and nutraceutical formulations

## 2. Materials and Methods

### 2.1. Torch Ginger Flower Samples

**Figure 1:**
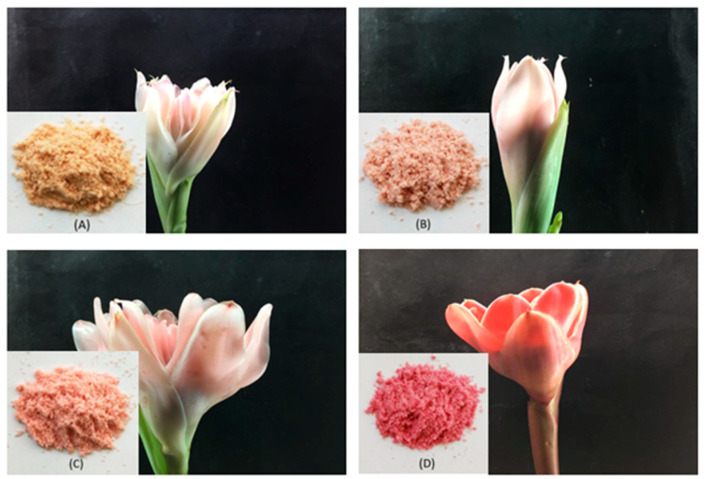
Torch ginger powdered samples from different types of color used for real sample application: (**A**) white, (**B**) faded pink, (**C**) pink, and (**D**) red.

The torch ginger (*Etlingera elatior*) flowers were obtained from the local store Dowa Kebun Organik, which is an organic garden located in Godean (Yogyakarta, Indonesia). The full-bloom flowers were harvested and then cleaned. For each flower type, approximately 70–80 individual torch ginger flowers were collected and pooled prior to processing in order to obtain representative samples and minimize variability among individual plants. The petals of the flowers were processed by freeze-drying for two days. A reduction in the size of the dried petals into powder was performed using a grinder. The fine-grained powder was then homogenized by stirring and shaking. The powdered samples were stored in an airtight container at 4 °C prior to analysis. Four different torch ginger flower samples, classified by their color, were used for the real sample application (Figure 1).

### 2.2. Chemicals

Methanol (HPLC-grade, assay 99.9%) was acquired from Panreac Quimica S.A.U. (Castellar del Vallés, Barcelona, Spain), and formic acid of analytical grade was obtained from Merck (Darmstadt, Germany). Ultrapure water was produced using a Milli-Q system (Millipore Corp., Bedford, MA, USA). To adjust the pH levels of the extraction solvent, hydrochloric acid (HCl) from Global Chem (Sevilla, Spain) was employed.

### 2.3. Ultrasound-Assisted Extraction

A Sonoplus HD 2070.2 processor (BANDELIN electronic GmbH and Co KG, Heinrichstraβe, Berlin, Germany) was used for the ultrasound-assisted extraction method. The system controls the cycle, amplitude, and working time. For the optimization step, the amplitude and cycle were set to 50% of the equipment’s maximum power (700 W) and 0.5 s^−1^, respectively, and the extraction time was 10 min. The temperature was controlled using an adjustable double vessel thermostatic bath (MX07R-20, San Diego, CA, USA). The UAE probe size used was approximately 130 mm in length and 13 mm in diameter (BANDELIN).

The weighed sample (approximately 1 g of a pool of real samples) was placed in a 50 mL falcon tube using an analytical balance (Mettler Toledo AG 135, Barcelona, Spain). Then, according to the experimental design, 10, 15, or 20 mL of solvent was added to the tube, and it was introduced into the double vessel thermostatic bath (which was at the right temperature according to the experiment) using a clamp to ensure it did not move during the extraction process. The pH of the solvent was adjusted to a value of 2 with hydrochloric acid (HCl) at a concentration of 1 M using a pH meter (Crison, GLP 22, Barcelona, Spain). The UAE probe was dipped 0.5 cm from the bottom of the tube, the amplitude and cycle were adjusted, and the extraction process was performed. Once extraction was complete, the sample was centrifuged at 4000 rpm for 10 min, and the solvent was then adjusted back to 10 mL. Subsequently, the extract was filtered through a 0.22 µm syringe filter (Nylon Syringe Filter, FILTER-LAB, Barcelona, Spain). Finally, the extracts were stored in a freezer at −20 °C for further analysis.

### 2.4. Anthocyanin Determination by Chromatographic Method

**Figure 2:**
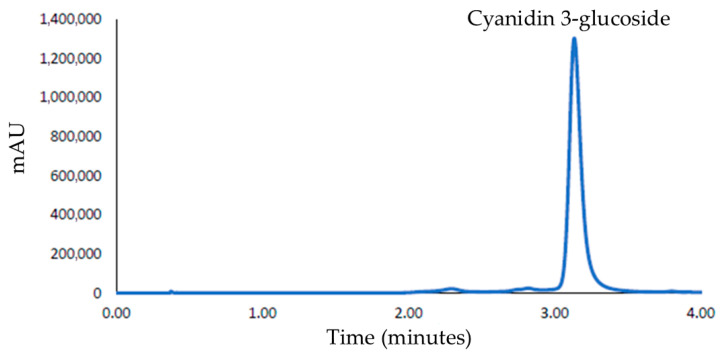
UPLC-Vis Chromatogram of anthocyanin separation from torch ginger sample.

An Elite UPLC LaChrom Ultra system (Hitachi, Tokyo, Japan) coupled with a UV–Vis L-2420U detector was used for anthocyanin determination. The same UPLC conditions used by Aliaño-González et al. were applied [33]. The system was equipped with an L-2200U autosampler to inject a 15 µL sample volume into the UPLC system. An L-2300 column oven set at 50 °C. Two L-2160 pumps were used to pump the mobile phase into the system at a flow rate of 0.7 mL min^−1^. The anthocyanins were separated in a reverse-phase C-18 (Phenomenex, Kinetex, CoreShell Technology, Torrance, CA, USA) of 2.1 × 50 mm and 2.6 µm particle size column. Gradient elution profiles for mobile phase A (5% formic acid in water) and B (100% methanol) were established to separate the anthocyanins in the sample, as follows: 0 min, 2% B; 1.5 min, 2% B; 3.3 min, 15% B; 4.8 min, 15% B; 5.4 min, 35% B; 6.0 min, 100% B. Both solvents were filtered through a 0.22 μm filter paper (GVS MAGNA nylon, Bolonia, Italy) to remove any contaminants and degassed using an Elmasonic S300 ultrasound system (Elma Schmidbauer GmbH, Singen, Germany) to eliminate excess gases that could interfere with the system. The chromatogram obtained after chromatographic analysis is shown in Figure 2.

The only anthocyanin identified in torch ginger flowers was cyanidin-3-glucoside (C3G), which was confirmed by the UPLC-PDA-QToF-MS system (Xevo G2 QToF, Waters Corp., Milford, MA, USA) (*m*/*z* = 449.1129). Thus, a calibration curve was constructed for anthocyanin quantification in the sample (regression equation: y = 168,154x – 28,462, LoD = 1.2 mg L^−1^, LoQ = 3.9 mg L^−1^; coefficient of determination: R^2^ = 0.9999).

### 2.5. Experimental Design for Extraction Optimization

Several factors were defined to optimize the extraction process. The design of experiment (DOE) was arranged using a Box–Behnken design (BBD) to reduce the number of runs in the experiment, leading to savings in solvents, time, and energy. Moreover, this approach avoids the need for experiments under extreme conditions and enables possible interaction effects between factors to be considered [34].

Statgraphic Centurion 18 (StatPoint Technologies, Inc., Warrenton, VA, USA) was used to build the experimental design and to analyze the data. The level chosen for each factor is described in Table 1.

The BBD was obtained from the four factors, which could have three possible values, coded as −1, 0, and 1, with 3 center points, so it consisted of 27 runs carried out in a random order to reduce the effect of uncontrolled factors that could have introduced bias into the measurements. The objective was to identify the optimal UAE conditions to obtain the highest concentration of anthocyanins. Finally, the results obtained by conducting the extraction under optimum conditions were compared with the predicted optimum response. The optimized extraction conditions were also validated.

The BBD approach was used to determine the influence of each factor and the interactions between factors, and to obtain the surface response by fitting the data to a second-order polynomial model [35].

$$y=\beta_{0}+\sum_{i=1}^{k}\beta_{i}\cdot x_{i}+\beta_{ii}\cdot x_{i}^{2}+\sum_{i}\sum_{i=1}^{k}\beta_{ij}\cdot x_{i}x_{j}+r$$

where *y* is the response; β_0_ is the constant coefficient; βi, βii, and βij are the regression coefficients of linear, quadratic, and interactive terms, respectively; *x* represents each factor; and *r* is the residual value.

### 2.6. Method Validation of Extraction Technique

Validation characteristics were evaluated by precision (intra-day and inter-day precision) and accuracy (recovery study) using the chromatographic method, because more reliable results were obtained that better fit the model, as will be explained in the results and discussion section.

Repeatability (intra-day precision) and intermediate precision (inter-day precision) were evaluated using replicate extractions performed on the same day and on different days. Accuracy was assessed through recovery studies using spiked samples. In this study, the spiking method was used to calculate the levels of analytes added to the sample matrix analyzed using the area of the peaks. As the standard solution for anthocyanins was not widely available commercially, a concentrated sample was used for the spiking method. Two different added concentrations (25% and 50% addition) of a concentrated sample were employed. The recovery evaluation was performed by comparing the concentrations of both spiked samples to the added concentration from the concentrated sample using the information on the total area after the extraction process, using the following equation:

% Recovery = (Ch − Cb)/Cs × 100%

where Ch is the spiked sample area (sample + concentrated sample); Cb is the sample area; and Cs is the standard area (concentrated sample).

### 2.7. Real Sample Application

The validated extraction method was applied to four torch ginger flower samples with different color intensities to evaluate its applicability to real food-related matrices. Extracts were analyzed in duplicate, and anthocyanin content was quantified to assess variability among samples.

This application demonstrates the suitability of the developed method for distinguishing anthocyanin levels in edible flowers with different pigmentation, supporting its use in ingredient selection and food product development.

## 3. Results and Discussion

### 3.1. Development of the UAE Method

The extraction method was optimized from the DOE result using chromatographic analysis. Response values corresponding to relative chromatographic peak areas normalized to the maximum area were obtained, which was observed in experiment #18.

The 27 experiments indicated by the BBD experimental design based on the factors analyzed, together with both responses obtained, that is, the relative values to the maximum total concentration of anthocyanins (%), are shown in Table 2.

**Figure 3:**
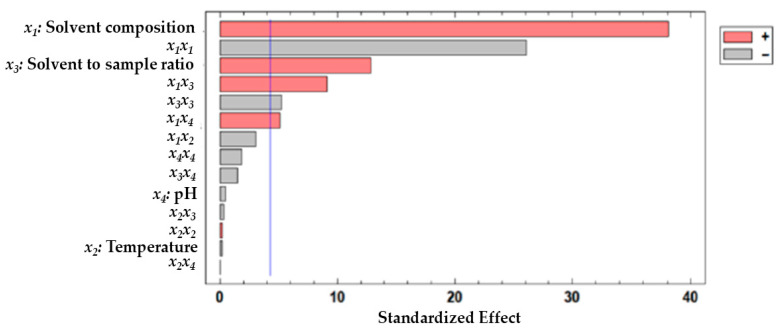
Standardized Pareto chart for anthocyanin extraction by UAE.

Analysis of variance (ANOVA) was performed to evaluate the effect of extraction factors and any possible interactions between them. Standardized Pareto charts were built to assess the statistical significance of the model by presenting the standardized effects of the main factors, interactions, and quadratic effects in decreasing order of significance. The factors or interactions that had a significant effect on the responses showed standardized values larger than 4.2 (as indicated by the vertical line in Figure 3).

This Figure shows that the variables have a significant effect on the UAE. The results indicate that solvent composition (*X*_1_) and solvent-to-sample ratio (*X*_3_) are the two most significant factors influencing the yield. The four significant interaction effects from this response were *X*_1_*X*_1_, *X*_1_*X*_3_, *X*_3_*X*_3_, and *X*_1_*X*_4_. In addition, the color of the bars refers to a direct or inverse relationship between each factor or interaction and the response.

The suitability of the fitted model was also evaluated by ANOVA. Based on the results (Table 3), the coefficient of determination (R^2^) was 96.62%. This value shows how well the model explains the variability of the response studied. These values are consistent with a statistically significant agreement between the measured and predicted responses. Additionally, the lack-of-fit test in the ANOVA table determines whether the model adequately describes the observed data or if a more complex model should be employed. The model appears to be adequate for the observed data at the 95% confidence level, as the *p*-value for lack-of-fit in the ANOVA table is greater than or equal to 0.05.

Once again, the variables or interactions that are significant in the response can be observed in this table.

### 3.2. Effects of UAE Factors

Based on the chromatographic analysis data results, the extraction yield was significantly affected by the solvent composition, with a positive effect, indicating that using more methanol will result in a higher concentration of anthocyanins in the extract. Methanol is often used as a solvent to extract anthocyanin compounds and has been found to be effective in extracting polyphenols with lower molecular weight. In the study of anthocyanin extraction from dried blackcurrants, anthocyanin concentration showed relatively higher concentrations when methanol in water was applied [36].

According to the mass transfer principle, the higher the solvent-to-sample weight ratio, the greater the overall amount of extract recovered. The driving force behind the extraction process comes from the concentration gradient between the solid and the solvent [37]. Because of the high solvent-to-solid ratio, the solvent can easily penetrate the target material’s cell walls and dissolve the target analytes. This is similar to the results of mulberry anthocyanin extraction, which confirmed that the solvent/solid ratio positively affected the extraction of anthocyanins [38]. Aslan Türker & Doğan (2022) also reported on their investigation of the ultrasound-assisted extraction of anthocyanin from a black carrot, wherein the solid/solvent ratio significantly impacted the amount of cyanidin-3-*O*-glucoside with a positive effect [39].

Furthermore, some interaction factors also appeared to be significant; the quadratic effect of solvent composition and the quadratic effect of the solvent-to-sample ratio have been found to have a negative effect, the interaction of solvent composition and solvent-to-sample ratio has a positive effect, and the interaction of solvent composition and pH also has a positive effect. The temperature and pH did not significantly affect the extraction yield, meaning that anthocyanins can be extracted from torch ginger using the UAE method at any temperature and pH without encountering many problems. Chen et al. (2020) carried out the optimization of ultrasound-assisted extraction of Rubia sylvatica Nakai fruit and found, as in this research, that pH and temperature did not significantly influence the extraction of total anthocyanins [40].

After the experiments had been carried out, the empirical regression model of the relationship between responses, including all factors in the second-order polynomial equation for the fitted model, was established in the following mathematical models, which can reliably predict the experimental results using chromatographic analysis:

Y_TA_ = 89.0435 + 20.9709*x*_1_ − 0.100635*x*_2_ + 7.06642*x*_3_ − 0.265554*x*_4_ − 21.5422*x*_1_*x*_1_ − 2.90776*x*_1_*x*_2_ + 8.70974*x*_1_*x*_3_ + 4.8554*x*_1_*x*_4_ + 0.184614*x*_2_*x*_2_
*−* 0.301779*x*_2_*x*_3_ − 0.0194578*x*_2_*x*_4_ − 4.29898*x*_3_*x*_3_ − 1.39958*x*_3_*x*_4_ − 1.50538*x*_4_*x*_4_

### 3.3. UAE Optimum Condition

**Figure 4:**
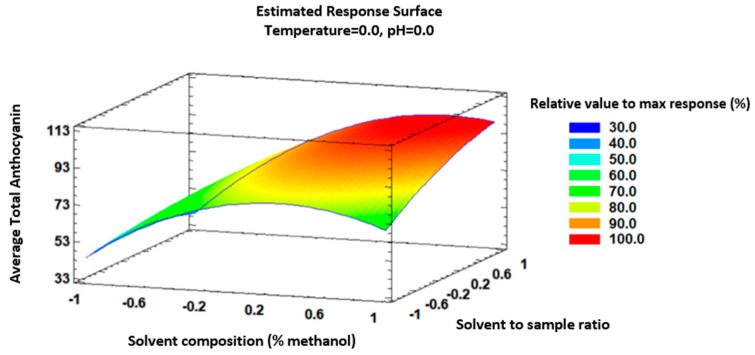
Response surface analysis of the extraction of total anthocyanins by means of UAE with respect to solvent composition and solvent-to-sample ratio.

A three-dimensional response surface plot was constructed to predict the relationship between the independent factors and responses (Figure 4). The optimum values were determined based on the predicted optimum total anthocyanin response in the UAE method: a solvent composition of 84% methanol in water as a solvent (predicted value 83.91%), a temperature of 30 °C (predicted value of 30 °C), a 20:1 ratio of solvent to sample in mL/g (predicted value of 19.7:1), and a pH of 5.6 (predicted value of 5.6).

Firstly, the percentage of methanol was determined by the polarity of both the compounds to be analyzed and the solvent [41]. A relatively high value was obtained, which indicates that the extracted anthocyanins are moderately polar and are therefore better dissolved in solvents containing a higher percentage of methanol than water.

On the other hand, it can be observed that for some of the factors, the extreme value of the analyzed range was obtained as the optimal value. The optimum temperature is the minimum of the range, which is closely correlated with the possibility of degrading these compounds at higher temperatures. Liu et al. (2026) used a temperature of 35 °C, as in this study, since preliminary trials confirmed that this temperature minimized anthocyanin degradation [42]. Regarding the ratio, the maximum volume of the studied range was the optimal value, due to the greater concentration gradient. However, no further tests with a greater volume of solvent were performed, since the compounds would be difficult to quantify if they were below their quantification limits [43].

Finally, the optimum value for pH was 5.6. According to Li et al. (2020), in the range of pH 3.00–6.00, the anthocyanin content kept rising until it reached a maximum of around pH 6. [44].

The optimum conditions were verified by conducting the experiments under the mentioned conditions. The estimation was that a total of 105.21% anthocyanin would be obtained relative to the maximum total anthocyanin obtained in the experiment design (5.21% higher than the maximum total anthocyanin values in the BBD results). As a result of the verification trials, the total anthocyanin was 14.59 ± 3% higher than the predicted value. Even though the results were higher than the values predicted by the model, the optimum UAE condition achieved a positive addition to the maximum total anthocyanin extracted.

### 3.4. UAE Kinetic Study

**Figure 5:**
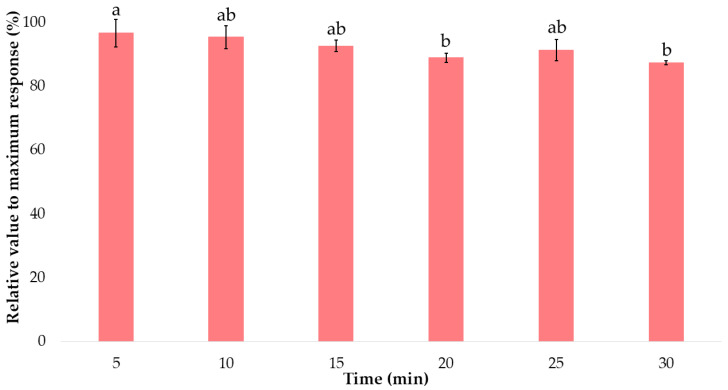
Optimization of UAE time. The same letter above each bar indicates that there were no significant differences in anthocyanin content according to Tukey’s test (*p*-value > 0.05).

Kinetic modeling was conducted to determine the optimal extraction time for anthocyanins in torch ginger. Several extractions were run under optimized UAE conditions at extraction times of 5, 10, 15, 20, 25, and 30 min. The results for the total anthocyanins in the sample are represented in Figure 5 using the average of the relative value of the highest total anthocyanins extracted. The graph showed that 5 min produced the highest recovery level in the extraction. In addition, there was no statistically significant difference between this and the extraction times of 10 and 15 min (with a lower standard deviation). Thus, 5 min extraction time was selected as optimal for extracting anthocyanins from the torch ginger flower samples. In general, a longer extraction time would lead to a decrease in the total anthocyanins. Similarly, the results of the kinetic study of anthocyanins in onion bulbs by V. González de Peredo et al. (2021) [45] demonstrated that after 5 min, the amount of anthocyanins extracted remained essentially the same, and that extraction for longer than 10 min led to lower extraction yields due to the degradation of the anthocyanin compounds. This result is particularly relevant for food applications, as shorter processing times reduce energy consumption and help preserve pigment functionality.

### 3.5. Validation Method

The parameters analyzed to validate the method were calculated by the total area of anthocyanin from UPLC-Vis chromatograms.

According to the AOAC (Association of Official Analytical Chemists) guidelines, the precision (repeatability and intermediate precision) is expressed as % CV. The acceptable CV limit was ±10%, according to the AOAC manual for the Peer-Verified Methods Program [46]. The repeatability and intermediate precision of the optimized UAE method were 5.6% and 4.6%, respectively. Both CV values indicate a precise extraction method, as they were below 10%.

The results of recovery by the UAE method were 92.6% and 88.9% for the 25% and 50% additions, respectively. According to the recommended acceptance criteria for analytical methods, the mean recovery should be within 80 and 110% of the target concentration [47]. Therefore, the recovery values were acceptable. From a food science perspective, method robustness is essential to ensure the consistent evaluation of raw materials and to support decision-making during ingredient selection and process optimization. The validated performance of the proposed method strengthens its suitability for such applications.

### 3.6. Real Sample Application of Optimized Extraction Method

The developed and validated UAE method was evaluated for its applicability to the extraction of anthocyanins in several real samples. Specifically, four different torch ginger flower samples based on their color (white, faded pink, pink, and red) were extracted in duplicate.

The extracts were analyzed by UPLC-Vis for the quantification of the total anthocyanin present in the samples (Table 4).

The UAE method demonstrated good applicability to real plant matrices, providing quantifiable levels of anthocyanins in all four torch ginger flower samples. As expected, the anthocyanin content clearly increased with flower color intensity. The white sample showed the lowest concentration (≈9 mg/100 g), whereas the red flower contained higher anthocyanin levels by an order of magnitude (≈178 mg/100 g).

This strong color–anthocyanin correlation is consistent with the known role of anthocyanins as pigments responsible for red, purple, and blue coloration in plant tissues. The trend confirms that the extraction method is sufficiently sensitive to detect compositional differences among samples. This finding is consistent with the results reported by Vizzotto et al. (2006) [48], who analyzed the anthocyanin content of 53 different varieties of plum fruit. These researchers confirmed that anthocyanin content tended to be higher in red/purple-flesh varieties as compared to those with light colored flesh.

The higher variability observed in the red samples could be related to natural heterogeneity in the plant material (e.g., maturity and tissue distribution), given that highly pigmented tissues often show greater biological variability.

## 4. Conclusions

This study demonstrates the suitability of ultrasound-assisted extraction as a rapid, efficient, and sustainable approach for the recovery of anthocyanins from torch ginger flowers, an edible plant matrix with growing relevance in food science. The optimized extraction conditions enabled high anthocyanin yields while minimizing processing time and thermal stress, two critical aspects for preserving the functionality and stability of natural pigments intended for food applications.

The ultrasound-assisted extraction method developed in this study was highly efficient for recovering anthocyanins from torch ginger flowers, offering short extraction times and minimal degradation of thermolabile compounds. The optimized UAE conditions consisted of 84% methanol at 30 °C, a 20:1 mL:g solvent-to-sample ratio, and a pH of 5.6. When applied to different flower color varieties, the method effectively distinguished their anthocyanin levels, revealing a clear correlation between pigment intensity and anthocyanin content, with the red variety showing the highest concentration (177.64 mg/100 g). Overall, this work provides the first validated UAE approach tailored to torch ginger flowers, offering a practical, fast, and environmentally friendly tool that supports their scientific characterization and enhances their potential application as natural colorants and functional food ingredients.

## Figures and Tables

**Table 1:** The chosen level for the variables of UAE.

Variables	Low	Center	High
	−1	0	+1
Solvent composition (%Methanol)	10	50	90
Temperature (°C)	30	50	70
Solvent-to-sample ratio (mL:g)	10:1	15:1	20:1
pH	2	4	6

**Table 2:** Box–Behnken design for UAE with observed responses.

Experiment Number	Solvent Composition (*x*_1_)	Temperature (*x*_2_)	Solvent-to-Sample Ratio (*x*_3_)	pH (*x*_4_)	Relative Values to the Maximum Area (%) in the Chromatographic Analysis
1	−1	0	1	0	33.67
2	1	0	1	0	97.39
3	0	0	0	0	91.17
4	0	1	0	−1	82.77
5	0	0	1	−1	89.83
6	0	0	0	0	88.46
7	−1	1	0	0	52.64
8	1	−1	0	0	88.33
9	1	1	0	0	88.60
10	−1	0	0	−1	57.94
11	0	−1	0	1	88.21
12	−1	−1	0	0	40.73
13	0	1	1	0	94.89
14	0	0	−1	1	79.23
15	0	−1	−1	0	79.09
16	0	−1	0	−1	84.92
17	−1	0	0	1	41.05
18	0	−1	1	0	100.00
19	1	0	0	1	88.50
20	0	1	−1	0	75.19
21	0	1	0	1	85.98
22	1	0	0	−1	85.96
23	1	0	−1	0	70.81
24	0	0	1	1	89.36
25	−1	0	−1	0	41.93
26	0	0	0	0	87.50
27	0	0	−1	−1	74.10

**Table 3:** Analysis of variance (ANOVA) of the quadratic model of total anthocyanins for chromatographic analysis in the UAE.

Source	Sum of Squares	Df	Mean Square	F-Ratio	*p*-Value
*X*_1_: Solvent composition	5277.32	1	5277.32	1452.28	0.0007
*X*_2_: Temperature	0.121528	1	0.121528	0.03	0.8718
*X*_3_: Solvent-to-sample ratio	599.211	1	599.211	164.9	0.006
*X*_4_: pH	0.84623	1	0.84623	0.23	0.6771
*X*_1_ *X*_1_	2475.02	1	2475.02	681.1	0.0015
*X*_1_ *X*_2_	33.8204	1	33.8204	9.31	0.0927
*X*_1_ *X*_3_	303.438	1	303.438	83.5	0.0118
*X*_1_ *X*_4_	94.2998	1	94.2998	25.95	0.0364
*X*_2_ *X*_2_	0.181772	1	0.181772	0.05	0.8438
*X*_2_ *X*_3_	0.364282	1	0.364282	0.1	0.7815
*X*_2_ *X*_4_	0.00151443	1	0.001514	0	0.9856
*X*_3_ *X*_3_	98.5665	1	98.5665	27.12	0.0349
*X*_3_ *X*_4_	7.83533	1	7.83533	2.16	0.2797
*X*_4_ *X*_4_	12.0862	1	12.0862	3.33	0.2098
Lack-of-fit	315.194	10	31.5194	8.67	0.1077
Pure error	7.26766	2	3.63383		
Total (corrected)	9534.81	26			

**Table 4:** Real sample application of UAE method.

Torch Ginger Flower Sample	Anthocyanin Content (mg C3G Equivalent/100 g)
White flower	9.41 ± 0.71
Fade-pink flower	18.51 ± 0.20
Pink flower	18.76 ± 0.22
Red flower	177.64 ± 19.34

## Data Availability

The original contributions presented in the study are included in the article, further inquiries can be directed to the corresponding author.

## Abbreviations

The following abbreviations are used in this manuscript:

UAE	Ultrasound-Assisted Extraction
UHPLC-DAD	Ultra high performance liquid chromatography—Photodiode array detector
CV	Coefficient of Variation
BBD	Box–Behnken Design
DOE	Design of Experiment
ANOVA	Analysis of Variance
AOAC	Association of Official Analytical Chemists
C3G	Cyanidin-3-Glucoside
